# Raisin allergy in an 8 year old patient

**DOI:** 10.1186/1710-1492-10-S2-A6

**Published:** 2014-12-18

**Authors:** S Chibuluzo, T Pitt

**Affiliations:** 1 St. George’s University, School of Medicine, Grenada, West Indies; 2 Humber River Hospital, Toronto, Ontario, Canada

## Introduction

Raisin allergy is uncommon despite worldwide cultivation of grapes, which belong to the *Vitis vinifera* species of the Vitaceae family. There have only been rare reports of anaphylaxis in adults related to the consumption of grapes, wine or other grape products, mostly in Europe [1-4]. In children, even fewer case reports to grape exist [5]. We report an 8-year-old patient who developed itching of the mouth and nausea within a few minutes of ingestion of fresh raisin on repeated occasions. Interestingly, he tolerates grapes.

## Methods

Skin prick testing (via prick-by-prick method) to fresh seedless raisin, birch pollen, a mixture of trees, grass, and ragweed was performed on the patient. Skin prick testing to fresh seedless raisin (via prick-by-prick method) was also performed on a non-atopic healthy control.

## Results

Skin testing was positive to fresh seedless raisins (~5 mm) in our 8-year-old patient and negative in the healthy control. The patient was advised to avoid raisins and to carry an Epinephrine auto-injector. He was encouraged to continue to consume fresh grapes.

## Conclusions

We report one of the first cases of presumed allergy to fresh raisin, in the absence of pollen food syndrome, in a North American patient who currently tolerates fresh grapes. Further research is required to determine the etiology and prevalence of this allergy. We propose that a chemical component used in the processing of raisins may be responsible for this allergy.

## Consent

Written informed consent was obtained from the patient for publication of this abstract and any accompanying images. A copy of the written consent is available for review by the Editor of this journal.

## References

[B1] Alcoceba Borràs E Botey Faraudo E Gaig Jané P Bartolomé Zavala B Alcohol-induced anaphylaxis to grapes Allergol Immunopathol (Madr) 2007 35 4 159 61 10.1157/13108228 17663926

[B2] Kalogeromitros DC Makris MP Gregoriou SG Mousatou VG Lyris NG Tarassi KE Papasteriades CA Grape anaphylaxis: a study of 11 adult onset cases Allergy Asthma Proc 2005 26 1 53 8 15813289

[B3] Caiaffa MF Tursi A Macchia L Grape anaphylaxis J Investig Allergol Clin Immunol 2003 13 3 211 2 14635473

[B4] Senna G Mistrello G Roncarolo D Crivellaro M Bonadonna P Schiappoli M Passalacqua G Exercise-induced anaphylaxis to grape Allergy 2001 56 12 1235 6 10.1034/j.1398-9995.2001.00352.x 11736764

[B5] Cardinale F Berardi M Chinellato I Damiani E Nettis E A child with anaphylaxis to grapes without reaction to grape seed oil Allergy 2010 65 6 800 1 19886916 10.1111/j.1398-9995.2009.02247.x

